# Supplementary material for: Does the COVID-19 pandemic lockdown affect risk attitudes?—Evidence from rural Thailand
Source: PLoS One. 2023 Oct 19;18(10):e0292873. doi: 10.1371/journal.pone.0292873 (PMC10586670; doi:10.1371/journal.pone.0292873)
Supplement: S1 Table — (PDF) [file pone.0292873.s001.pdf]

S1 Table. Overview of studies investigating the effects of the COVID-19 pandemic on risk attitudes

Authors	Sampling	Measures of risk attitudes	Changes in risk aversion
Lohmann et al. [1]	522 Chinese students	Gamble choice task [2]; Investment game [3]	stable
Drichoutis and Nayga [4]	495 Greek students	Gamble choice task [5];	stable
Angrisani et al. [6]	48 professional traders and 60 students in London	Balloon Analogue Risk Task (BART) [7]	stable
Shachat et al. [8]	396 Chinese students	Gamble choice task [5]	decrease
Li [9]	294 Chinese undergraduate students	Domain-specific risk-attitude scale [10]	decrease
Tsai and Zeng [11]	661 MTurk participants in the U.S. and 234 students in Canada	Domain-specific risk-attitude scale [10]; General willingness to take risks [12]	decrease
Tsutsui and Tsutsui-Kimura [13]	4,359 residents in Japan	Willingness to pay for insurances	decrease
Castillo and Hernandez [14]	1,824 agricultural households in Guatemala	General willingness to take risks [12]	decrease
Gassmann et al. [15]	128 French students	Multiple Price List format	decrease, but effects attenuate over time
Ikeda et al. [16]	2,894 Japanese	Hypothetical questions about insurance purchase	decrease in the loss domain
Adema et al. [17]	606 students in Czachia, India, Mexico and Spain	General willingness to take risks & gamble choice task [18]	increase using self-assessment; decrease using the gamble choice task
Bu et al. [19]	225 Chinese students	Hypothetical lottery question; survey-based measure [18]	increase
Li et al. [20]	585 Chinese students	Gamble choice task [5]	increase
Mussio et al. [21]	1,408 MTurk participants in the U.S.	General willingness to take risks [12]; Gamble choice task [22]	increase
Alsharawy et al. [23]	500 MTurk participants in the U.S.	General willingness to take risks [12]; Gamble choice task [2]	increase
Graeber et al. [24]	6,700 Germans	General willingness to take risks [12]	increase
Frondel et al. [25]	5,165 German household heads	General willingness to take risks [12]	increase

Authors	Sampling	Measures of risk attitudes	Changes in risk aversion
Heo et al. [26]	18,193 U.S. financial decision-makers	Financial risk tolerance assessment [27]	increase
Zhang and Palma [28]	around 80 MTurk participants in each round of the experiment	Balloon Analogue Risk Task (BART) [7]; Gamble choice task [2]; Domain-specific risk-taking scale [29]; Sensation-seeking scale [30]	increase for men using BART; stable using other measures
Huber et al. [31]	315 financial professionals in Europe and 498 German students	Hypothetical investment task	increase for financial professionals; stable for students

References

1. Lohmann PM, Gsottbauer E, You J, Kontoleon A. Anti-social behaviour and economic decision-making: Panel experimental evidence in the wake of COVID-19. J Econ Behav Organ. 2023 Feb 1;206:136–71.

2. Eckel CC, Grossman PJ. Sex differences and statistical stereotyping in attitudes toward financial risk. Evol Hum Behav. 2002 Jul 1;23[4]:281–95.

3. Gneezy U, Potters J. An experiment on risk taking and evaluation periods. Q J Econ. 1997 May 1;112[2]:631–45.

4. Drichoutis AC, Nayga RM. On the stability of risk and time preferences amid the COVID-19 pandemic. Exp Econ. 2022 Jun 1;25[3]:759–94.

5. Holt CA, Laury SK. Risk Aversion and Incentive Effects. Am Econ Rev. 2002 Dec;92[5]:1644–55.

6. Angrisani M, Cipriani M, Guarino A, Kendall R, Ortiz de Zarate J. Risk preferences at the time of COVID-19: An experiment with professional traders and students [Internet]. Rochester, NY: Rochester, NY; 2020 May [cited 2023 Feb 11]. Report No.: 3609586. Available from: https://papers.ssrn.com/abstract=3609586

7. Lejuez CW, Read JP, Kahler CW, Richards JB, Ramsey SE, Stuart GL, et al. Evaluation of a behavioral measure of risk taking: The Balloon Analogue Risk Task [BART]. J Exp Psychol Appl. 2002;8:75–84.

8. Shachat J, Walker MJ, Wei L. How the onset of the Covid-19 pandemic impacted pro-social behaviour and individual preferences: Experimental evidence from China. J Econ Behav Organ. 2021 Oct 1;190:480–94.

9. Li H. Life is either a daring adventure, or it is boring: The impact of COVID-19 on immoral and nonmoral risk taking behaviors. J Behav Decis Mak. 2023;e2319.

10. Weber EU, Blais AR, Betz NE. A domain-specific risk-attitude scale: measuring risk perceptions and risk behaviors. J Behav Decis Mak. 2002;15[4]:263–90.

11. Tsai CI, Zeng Y. Risky but alluring: Severe COVID-19 pandemic influence increases risk taking. J Exp Psychol Appl. 2021;27[4]:679–94.

12. Dohmen T, Huffman D, Schupp J, Falk A, Sunde U, Wagner GG. Individual risk attitudes: Measurement, determinants, and behavioral consequences. J Eur Econ Assoc. 2011;9[3]:522–50.

13. Tsutsui Y, Tsutsui-Kimura I. How does risk preference change under the stress of COVID-19? Evidence from Japan. J Risk Uncertain. 2022 Apr 1;64[2]:191–212.

14. Castillo JG, Hernandez MA. The unintended consequences of confinement: Evidence from the rural area in Guatemala. J Econ Psychol. 2023 Mar 1;95:102587.

15. Gassmann X, Malézieux A, Spiegelman E, Tisserand JC. Preferences after pan[dem]ics: Time and risk in the shadow of COVID-19. Judgm Decis Mak. 2022;17[4]:23.

16. Ikeda S, Yamamura E, Tsutsui Y. COVID-19 enhanced diminishing sensitivity in prospect-theory risk preferences: A panel analysis [Internet]. Rochester, NY: Rochester, NY; 2020 Oct [cited 2023 Feb 13]. Report No.: 3715236. Available from: https://papers.ssrn.com/abstract=3715236

17. Adema J, Nikolka T, Poutvaara P, Sunde U. On the stability of risk preferences: Measurement matters. Econ Lett. 2022 Jan 1;210:110172.

18. Falk A, Becker A, Dohmen T, Enke B, Huffman D, Sunde U. Global evidence on economic preferences. Q J Econ. 2018 Nov 1;133[4]:1645–92.

19. Bu D, Hanspal T, Liao Y, Liu Y. Risk taking, preferences, and beliefs: Evidence from Wuhan [Internet]. Rochester, NY: Rochester, NY; 2020 Dec [cited 2023 Feb 13]. Report No.: 3559870. Available from: https://papers.ssrn.com/abstract=3559870

20. Li Z, Lin PH, Kong SY, Wang D, Duffy J. Conducting large, repeated, multi-game economic experiments using mobile platforms. PLOS ONE. 2021 Apr 29;16[4]:e0250668.

21. Mussio I, Sosa Andrés M, Kidwai AH. Higher order risk attitudes in the time of COVID-19: an experimental study. Oxf Econ Pap. 2023 Jan 1;75[1]:163–82.

22. Eeckhoudt L, Schlesinger H. Putting Risk in Its Proper Place. Am Econ Rev. 2006 Mar;96[1]:280–9.

23. Alsharawy A, Ball S, Smith A, Spoon R. Fear of COVID-19 changes economic preferences: evidence from a repeated cross-sectional MTurk survey. J Econ Sci Assoc. 2021 Dec 1;7[2]:103–19.

24. Graeber D, Schmidt U, Schroeder C, Seebauer J. The effect of a major pandemic on risk preferences - Evidence from exposure to COVID-19 [Internet]. Rochester, NY: Rochester, NY; 2020 Nov [cited 2023 Feb 13]. Report No.: 3724461. Available from: https://papers.ssrn.com/abstract=3724461

25. Frondel M, Osberghaus D, Sommer S. Corona and the stability of personal traits and preferences: Evidence from Germany [Internet]. Mannheim, Germany: Leibniz-Zentrum für Europäische Wirtschaftsforschung [ZEW], Mannheim, Germany; 2021 [cited 2023 Feb 13]. Report No.: 21–029. Available from: https://www.econstor.eu/handle/10419/232943

26. Heo W, Rabbani A, Grable JE. An Evaluation of the Effect of the COVID-19 Pandemic on the Risk Tolerance of Financial Decision Makers. Finance Res Lett. 2021 Jul 1;41:101842.

27. Grable J, Lytton RH. Financial risk tolerance revisited: the development of a risk assessment instrument☆. Financ Serv Rev. 1999 Jan 1;8[3]:163–81.

28. Zhang P, Palma MA. Stability of Risk Preferences During COVID-19: Evidence From Four Measurements. Front Psychol [Internet]. 2022 [cited 2023 Aug 14];12. Available from: https://www.frontiersin.org/articles/10.3389/fpsyg.2021.702028

29. Blais AR, Weber EU. A Domain-Specific Risk-Taking [DOSPERT] scale for adult populations. Judgm Decis Mak. 2006 Jul;1[1]:33–47.

30. Zuckerman M, Kolin EA, Price L, Zoob I. Development of a sensation-seeking scale. J Consult Psychol. 1964;28[6]:477–82.

31. Huber C, Huber J, Kirchler M. Market shocks and professionals' investment behavior – Evidence from the COVID-19 crash. J Bank Finance. 2021 Dec 1;133:106247.
