# Supplementary material for: Does the COVID-19 pandemic lockdown affect risk attitudes?—Evidence from rural Thailand
Source: PLoS One. 2023 Oct 19;18(10):e0292873. doi: 10.1371/journal.pone.0292873 (PMC10586670; doi:10.1371/journal.pone.0292873)
Supplement: S2 Table — (PDF) [file pone.0292873.s002.pdf]

S2 Table. Variable description

Variable	Description
Panel A. Individual level	
Willingnes to take risk	Measured by the question “Are you generally a person who is fully prepared to take risks or do you try to avoid taking risks?”, answering on a scale of 0-10, where 0 means “unwilling to take risks” and 10 means “fully prepared to take risks”
Age	Age in years
Female	Dummy variable equals 1 for females; 0 for males
Married	Dummy variable equals 1 for being married; 0 otherwise
Education (years)	Education in years
Agricultural occupation	Dummy variable equals 1 for working in the agricultural sector; 0 for working outside the non-agricultural sector
Income fluctuation	Dummy variable equals 1 if there is a bit or a lot of fluctuation in income; 0 if there is no fluctuation in household income at all
Household size	Household size
Dependency ratio	Calculated as the ratio of the sum of the number of children under 15 and the number of persons aged 65 and above divided by the total number of persons in a household
Sick	Dummy variable equals 1 if the subject is sick; 0 if the subject is healthy or able to manage their health issues
Under- or overweight	Dummy variable equals 1 if the subject’s body mass index (BMI) <18.5 or BMI ≥ 25.0; 0 if $18.5 \leq$ BMI < 25.0
Handling stress	Measured by the question “Do you see yourself as someone who is relaxed, handles (the COVID-19 related) stress well?”, answering on a scale of 1-7, where 1 means “does not apply to me at all” and 7 means “applies to me perfectly”
Getting nervous	Measured by the question “(During the Covid-19 crisis,) Do you see yourself as someone who gets nervous easily?”, answering on a scale of 1-7, where 1 means “does not apply to me at all” and 7 means “applies to me perfectly”
COVID symptoms	Dummy variable equals 1 for respondents showing symptoms of COVID-19; 0 for respondents without symptoms
Financial impact of COVID	Dummy variable equals 1 for respondents reporting a negative or very negative impact of the COVID-19 crisis on the household’s financial situation; 0 for respondents reporting no impact, a positive impact or a very positive impact on the household's financial situation
Panel B. Village level	
Declaration of lockdown	Dummy variable equals 1 for villages with a declared lockdown; 0 for villages without a lockdown
Lockdown duration	Lockdown duration in days
Curfew	Dummy variable equals 1 for villages where a curfew was in place; 0 otherwise
No drinking parties	Dummy variable equals 1 for villages where drinking parties were not allowed; 0 otherwise
Closing schools	Dummy variable equals 1 for villages with school closure; 0 otherwise
Restricting visiting temples	Dummy variable equals 1 for villages where visiting temples was forbidden; 0 otherwise
