# Supplementary material for: Does the COVID-19 pandemic lockdown affect risk attitudes?—Evidence from rural Thailand
Source: PLoS One. 2023 Oct 19;18(10):e0292873. doi: 10.1371/journal.pone.0292873 (PMC10586670; doi:10.1371/journal.pone.0292873)
Supplement: Attachment — Submitted filename: reviwers comments.pdf [file pone.0292873.s003.pdf]

Reviewer's comments on "Does the COVID-19 pandemic lockdown affect risk attitudes? - Evidence from rural Thailand"

Abstract of this study: This study aims to examine how Covid-19 has changed the risk attitude of Thai residents. To find this out, the authors used 2019 and 2020 data of the research project "Poverty dynamics and sustainable development: A long-term panel project in Thailand and Vietnam, 2015 – 2024," to estimate how people's risk attitude changed by lockdown duration (day) in 19 out of 220 villages in 2020. They found that the coefficient of lockdown duration was negative but insignificant. Next, to allow for the possibility that this effect may vary by attribute, the authors included the intersection of various attributes and lockdown duration, and found that none of these were significant, except for the intersection with the agricultural worker dummy, which was significantly positive at 10%. The authors summarize these results as follows: "Our results provide further insights into the impact of shocks on rural households' risk attitudes. This sheds light on how policy designs can better help mitigate downward economic trends following exogenous shocks."

Major comments:

1. We observe risk taking behavior, though risk attitude (a type of preference) cannot be directly observed. Therefore, we must estimate the risk attitude from the observed risk-taking behavior. For example, in the arrow-Platt measure, the curvature of the utility function is estimated. Risk taking behavior is the choice between different risk options, which depends on the magnitude of the risks faced and the risk attitude of the individual. Some studies used measures for risk attitude that do not fully eliminate the change in risk, even though risk changes before and after the event (see papers on the 2008 financial crisis). I imagine that this has resulted in a mixed literature in which people have become risk averse and risk tolerant due to mega events. I imagine that the former is more prevalent because changes in risk cannot always be successfully ruled out. This paper reviews a number of papers, and I hope the authors will try to organize the literature from this perspective.

   It is a convenient assumption for economists for making theoretical predictions that risk attitude varies across individuals but is stable within individuals over time. Without it, economic theory would be too complex to make useful predictions. But this argument, of course, does not claim that people's risk-taking behavior is constant. People's choices change as the magnitude of risk changes, even if risk attitude is stable.
2. In this paper, self-evaluation of one's risk attitude or risk-taking behavior is used as an indicator of risk attitude. Whether this indicator represents risk attitude or risk-taking behavior still requires careful examination. In this sense, this study is evaluated as adding

another example to the confusing situation that the previous literature has presented.

3. This paper reports “negative results” meaning that the key variable of ‘Lockdown duration’ is not significant at all for most of estimations. Please discuss why the key variable was insignificant. I guess that small number of observations of positive duration, only 19 villages (8.3% of total villages), might be the cause. Another possibility is that situation of infection was not really changed by lockdown. If these suppositions are the case, ‘Lockdown duration’ is not an appropriate variable to investigate the effect of Covid-19.

   If the authors cannot offer a meaningful hypothesis that explains insignificant ‘Lockdown duration,’ it might be better to focus on 2020 year dummy, which was highly significant rather than ‘Lockdown duration.’ In this case, descriptive facts that Covid-19 was the largest event in Thailand in 2020 may be necessary.
4. The authors claim that the contribution of this study is to examine “the impact of the pandemic on risk attitudes of the rural poor in developing countries, a group which is particularly vulnerable to exogenous shocks.” If so, they may want to offer their hypothesis concerning the impact, and evaluate their results based on the hypothesis.

Minor comments:

5. ‘Handling stress’ and ‘Getting nervous’ seem to be opposite variables. Is it correct that they show the same sign in Table 2?
6. Please show more information, such as histogram, of ‘Lockdown duration’.
